# Effects of the Macular Carotenoid Lutein in Human Retinal Pigment Epithelial Cells

**DOI:** 10.3390/antiox6040100

**Published:** 2017-12-04

**Authors:** Xiaoming Gong, Christian S. Draper, Geoffrey S. Allison, Raju Marisiddaiah, Lewis P. Rubin

**Affiliations:** 1 Department of Pediatrics, Paul L. Foster School of Medicine, Texas Tech University Health Sciences Center, El Paso, TX 79905, USA; Xiaoming.gong@ttuhsc.edu; 2 Paul L. Foster School of Medicine, Texas Tech University Health Sciences Center, El Paso, TX 79905, USA; christian.draper@ttuhsc.edu (C.S.D.); g.allison@ttuhsc.edu (G.S.A.); 3 All Children’s Research Institute, St. Petersburg, FL 33701, USA; hmraju@gmail.com; 4 Department of Biomedical Sciences, Paul L. Foster School of Medicine, Texas Tech University Health Sciences Center, El Paso, TX 79905, USA

**Keywords:** xanthophyll, lutein, lycopene, carotenoids, retinal pigment epithelium, hypoxia, oxidative stress, tBHP

## Abstract

Retinal pigment epithelial (RPE) cells are central to retinal health and homoeostasis. Oxidative stress-induced damage to the RPE occurs as part of the pathogenesis of age-related macular degeneration and neovascular retinopathies (e.g., retinopathy of prematurity, diabetic retinopathy). The xanthophyll carotenoids, lutein and zeaxanthin, are selectively taken up by the RPE, preferentially accumulated in the human macula, and transferred to photoreceptors. These macular xanthophylls protect the macula (and the broader retina) via their antioxidant and photo-protective activities. This study was designed to investigate effects of various carotenoids (β-carotene, lycopene, and lutein) on RPE cells subjected to either hypoxia or oxidative stress, in order to determine if there is effect specificity for macular pigment carotenoids. Using human RPE-derived ARPE-19 cells as an in vitro model, we exposed RPE cells to various concentrations of the specific carotenoids, followed by either graded hypoxia or oxidative stress using *tert*-butyl hydroperoxide (tBHP). The results indicate that lutein and lycopene, but not β-carotene, inhibit cell growth in undifferentiated ARPE-19 cells. Moreover, cell viability was decreased under hypoxic conditions. Pre-incubation of ARPE-19 cells with lutein or lycopene protected against tBHP-induced cell loss and cell co-exposure of lutein or lycopene with tBHP essentially neutralized tBHP-dependent cell death at tBHP concentrations up to 500 μM. Our findings indicate that lutein and lycopene inhibit the growth of human RPE cells and protect the RPE against oxidative stress-induced cell loss. These findings contribute to the understanding of the protective mechanisms attributable to retinal xanthophylls in eye health and retinopathies.

## 1. Introduction

The retinal pigment epithelium (RPE) is a gatekeeper of the outer retina and controls the movement of nutrients, metabolites, and fluid between the outer retina and the choroidal blood supply. Additionally, the RPE cell layer is essential for maintaining retinal integrity and photoreceptor survival [1]. The RPE in situ is exposed to high levels of blue light and high oxygen tension, rendering it highly susceptible to oxidative stress. Oxidative damage to the RPE occurs as part of the pathogenesis of neovascular retinopathies, such as retinopathy of prematurity (ROP) and diabetic retinopathy (DR), which both are injury-induced (including oxidative damage) retinopathies arising in the retina vasculature-, and age-related macular degeneration (AMD) [2,3,4,5]. Specific xanthophyll carotenoids, namely lutein and zeaxanthin, are preferentially accumulated in the retina, and especially the macula, to comprise macular pigment and protect the macula from oxidative damage [6,7]. Lutein and zeaxanthin are present in human RPE, although at relatively lower levels as compared to neuroretinal layers [8,9]. However, the RPE controls nutrient and metabolite transport, including that of xanthophylls. Consequently, it is hypothesized that the accumulation and turnover of xanthophylls in the macula may be tightly regulated by the RPE, via as yet undetermined mechanisms.

Carotenoids, which are primarily plant-derived lipophilic pigments, are essential factors in human health and development. Specifically, they function in a wide range of biological processes, including reproduction, embryonic development, immunity and vision. Carotenoids may be divided into two general classes: carotenes and xanthophylls. Carotenes are non-polar molecules, which contain only carbon and hydrogen atoms, whereas xanthophylls are polar carotenoids, containing at least one oxygen atom [10]. In turn, xanthophylls may be subdivided into hydroxyl-carotenoids containing one or two hydroxyl groups and keto-carotenoids containing ketone groups. Of the >600 described natural carotenoids, approximately 50 are typically consumed in the human diet, but only 15–20 are routinely detectable in human serum and tissues, including lycopene, alpha-carotene, beta-carotene, beta-cryptoxanthin, lutein, and zeaxanthin [11]. Among this group, the xanthophylls (lutein and zeaxanthin) account for 20–30% of total carotenoids in human serum and 80–90% of total carotenoids in the human retina^6^. In early development, lutein is the predominant retinal carotenoid. Over time, zeaxanthin levels rise, partly due to conversion of lutein to zeaxanthin via meso-zeaxanthin formation. These xanthophylls are most dense at the center of the fovea in the yellowish pigmented area called the macula lutea and constitute macular pigment [7,12]. Lutein and zeaxanthin confer macular protection via antioxidant and light-screening properties [13]. An inverse association between macular pigment density and AMD has long been recognized [14,15]. However, despite these relationships, little is known about how the RPE regulates xanthophyll metabolism and macular pigment density under physiological and pathological retinal conditions.

The RPE is a highly metabolically active tissue layer that resides in a pro-oxidant, actinic stress environment, due to abundant light influx and exposure to high oxygen concentrations. Free oxygen radical formation in the retina is facilitated by the retina’s structural properties, including high oxygen concentration and dense blood supply, high concentrations of polyunsaturated fatty acids, and exposure to visible and UV light. In addition, the RPE phagocytoses and handles the debris from rapidly turning over photoreceptor outer segments (POS). This disproportionate burden of oxidative and metabolic stress contributes to the development of several retinal diseases. Numerous studies have demonstrated the anti-oxidative properties of retinal lutein and zeaxanthin [16,17] as well as functions in blue light filtering, regulating photo stress recovery time, and neural processing speed [17].

In this study, we primarily used undifferentiated human ARPE-19 cells to probe the protective response of dietary carotenoids—lutein, beta-carotene, and lycopene—against oxidative damage and hypoxic challenge. We analyzed the dynamic uptakes of lutein in the ARPE-19 cells and lutein metabolism-related gene expression, in response to selected carotenoid treatments. We show for the first time that lutein and lycopene, but not β-carotene, inhibit RPE cell growth and protect RPE cells from oxidative stress-induced cell death.

## 2. Materials and Methods

### 2.1. Materials

The human retinal pigment epithelial cell line, ARPE-19 cell line, was obtained from the American Type Culture Collection (ATCC, Rockville, MD, USA). Lutein was obtained from Kemin Health Inc (FloraGLO Lutein 10% VG TabGrade, Des Moines, IA, USA). Beta-carotene was obtained from Sigma–Aldrich (St. Louis, MO, USA). Lycopene was obtained from BASF (Ludwigshafen, Germany). Working solutions of carotenoids were freshly prepared with DMSO and tetrahydroflan (THF) in a ratio of 2:1, immediately before use for experiments. The tert-butyl hydroperoxide (tBHP) was purchased from Sigma–Aldrich.

### 2.2. Methods

#### 2.2.1. Cell Culture

ARPE-19 cells (ATCC CRL2302) were maintained in Dulbecco’s modified Eagle’s medium/F-12 nutrient medium (DMEM/F-12; Gibco BRL, Carlsbad, CA, USA), supplemented with 10% heat-inactivated fetal bovine serum (Gibco BRL) and 1% penicillin-streptomycin. Cells were kept under minimal light exposure in an incubator, at 37 °C and 5% CO_2_, in a humidified atmosphere, and passaged with 0.25% Trypsin–EDTA (Life Technologies, Carlsbad, CA, USA), every 3–4 days. RPE cells within the first 10 passages were selected and placed into appropriate culture plates for the experiments.

#### 2.2.2. Cell Treatments

Cell growth experiments were performed by plating ARPE-19 cells on 96-well plates at a concentration of 10,000 cells/mL/well in either serum-free or serum-containing medium. After washing with fresh serum-free medium, cells were treated, as indicated below. Untreated cells or cells treated with dimethyl sulfoxide (DMSO, Sigma–Aldrich, St. Louis, MO, USA) at a concentration identical to that present in the carotenoid-containing samples served as negative controls.

In hypoxia experiments, ARPE-19 cells were seeded into 24-well plates at a density of 1 × 10^5^ cells per well. Cells were incubated in a sealed chamber at 37 °C for the indicated times in a controlled environment of 1% O_2_, in the presence of 5% CO_2_ and 94% N_2_, using a PROOX 100 culture system (BioSpherix, Redfield, NY, USA). Cells cultured under standard conditions (21% O_2_, 5% CO_2_, and 74% N_2_) served as normoxic control cultures. All experiments were performed in triplicate.

#### 2.2.3. Determination the Uptake of Lutein

To determine the intake of lutein, confluent monolayers of ARPE-19 cells were incubated with lutein, in medium containing 10% FBS, in a time- and dose-dependent manner. Lutein was extracted as described previously, with minor modifications [18]. After incubation of ARPE-19 cells with known concentrations of lutein for the indicated time, the cell culture plates were placed on ice, the medium was removed, and monolayers were washed once with 0.5 mL of 10 mM sodium taurocholate in phosphate-buffered saline (PBS) to remove surface-bound carotenoids, followed by two additional PBS washings. The washed cells were harvested by brief trypsinization and the cell pellets were homogenized in 0.5 mL ice-cold PBS and transferred to glass tubes. An aliquot (0.1 mL from 0.2 mM stock) of butylated hydroxytoluene (BHT) was added to the homogenate. Lycopene extraction was performed by vigorous mixing with 1.5 mL dichloromethane/methanol (1:2, v/v) followed by 2 mL hexane. Following centrifugation, the resulting upper layer of hexane-dichloromethane was collected. The lower layer was similarly extracted two more times and the hexane-dichloromethane layers were pooled. The combined extract was dried under a Speedvac concentrator (Model: Savant AS160), re-dissolved in 0.1 mL dichloromethane/methanol (1:4, v/v), and subjected to HPLC analysis. We also analyzed the concentrations of lycopene in the medium, before and after incubations. Sample handling, homogenization, and extraction were carried out under a low temperature and dim yellow light to minimize isomerization.

#### 2.2.4. HPLC Analysis of Lutein

Lutein was analyzed and quantified, as described previously, with minor modifications [19]. A Shimadzu HPLC system (Model: UFLC, Kyoto, Japan) equipped with a PDA detector, SPD-M20A, monitoring from 210 to 670 nm, comprising a gradient pump system, LC-20AT, and a personal computer equipped with LC Solution software (Shimadzu), was used for the detection and quantification of lycopene. Lycopene was separated on a C30 carotenoid column (5 µm, 4.6 × 150 mm, YMC; Waters), attached to a guard cartridge (5 µm, 4.0 × 20 mm, YMC; Waters).

#### 2.2.5. Cell Viability Assays

ARPE-19 cells were seeded in 96-well plates at a density of 2 × 10^3^ cell/well and cultured in 100 μL medium overnight for attachment. Then, the culture medium was removed and the cells were treated with serial dilutions of carotenoids (0.5–2.0 μM) in fresh culture medium with 10% FBS or cultured in a medium with DMSO as a control. Cell viability was determined after 48 h by the 3-(4,5-dimethylthiazol-2-yl)-2,5-diphenyltetrazolium bromide (MTT) assay using the CellTiter96^®^ Non-Radioactive Cell Proliferation Kits (Promega, Madison, WI, USA), in which the yellow tetrazolium salt is reduced by mitochondrial dehydrogenase of viable cells to purple, insoluble crystals of formazan. Cells were incubated for 4 h with MTT solution at 37 °C. Then, formazan crystals were solubilized in lysing buffer overnight at room temperature (RT). The reaction product was quantified by measurement of absorbance at a 570 nm wavelength using a BioTek Synergy™ H4 Hybrid Multi-Mode Microplate Reader (BioTek Instruments Inc., Winooski, VT, USA). All experiments were performed in triplicate. Results are representative of an average of 3 independent experiments. Data are presented as proportional viability (%), by comparing the treated group with the untreated cells, the viability of which is measured to be 100%.

#### 2.2.6. qRT-PCR Using TaqMan RNA Assays and Data Analysis

Total RNA was extracted using RNeasy Mini Kits (Qiagen, Germantown, MD, USA) according to the manufacturer’s instructions and quantified using a Nanodrop 2000 UV-visible spectrophotometer. Quantitative real time PCR was carried out in triplicate using a Model 7500 fast real-time PCR system and the TaqMan method (Applied Biosystems, Foster City, CA, USA). TaqMan primers and probes for β-carotene 15,15′-oxygenase (BCO1, Hs00363176_ml), β-carotene 9′,10′-oxygenase (BCO2, Hs00230564_n1), scavenger receptor class B member 1 (Hs00969821_m1, SR-B1), cluster of differentiation 36 (Hs00354519_m1, CD36), low-density lipoprotein receptor (Hs01092524_m1, LDLR), ATP-binding cassette transporter A1 (Hs01059137_m1, ABCA1), vascular endothelial growth factor (Hs00900055_m1, VEGF), and glyceraldehyde-3-phosphate dehydrogenase (GAPDH, Hs02758991_g1) were purchased from Applied Biosystems. A quantity of 1 μg of total RNA was used in reverse transcription, and PCR was performed, according to the manufacturer’s protocol (Applied Biosystems, Foster, CA, USA). The expression level of targets was calculated using the Δ-Ct transformation method and data are shown as ratios after normalization to the constitutively expressed genes 18s rRNA and GAPDH. The qRT-PCR results were obtained from at least two independent experiments; qPCR data were analyzed using the comparative 2^−ΔΔCt^ method.

#### 2.2.7. Western Blotting

ARPE-19 cells were lysed and homogenized, as described previously. A quantity of 30 μg of total protein lysates was loaded onto NuPAGE 4–12% Tris-acetate gels (Invitrogen) for electrophoresis. Proteins were subsequently transferred onto PVDF membranes, using X Cell II™ Blot Module (Invitrogen, Carlsbad, CA, USA). Membranes were treated with casein blocker in PBS (Thermo Scientific, Waltham, MA, USA) for at least one hour at room temperature and probed with the appropriate primary antibody, followed by incubation with the appropriate anti-rabbit or anti-mouse IgG conjugated to horseradish peroxidase (Amersham Biosciences, Uppsala, Sweden). Proteins were detected using ECL™ Western blotting reagents (Amersham).

#### 2.2.8. Statistical Analysis

Experiments were conducted either in duplicate or in triplicate and all experiments were repeated at least three times. Data are expressed as means ± SD from at least three experiments. Student’s two-tailed *t* tests were used to evaluate the statistical significance of differences between groups; *p* < 0.05 was considered statistically significant.

## 3. Results

### 3.1. Lutein Uptake and Accumulation in ARPE-19 Cells

**Figure 1:**
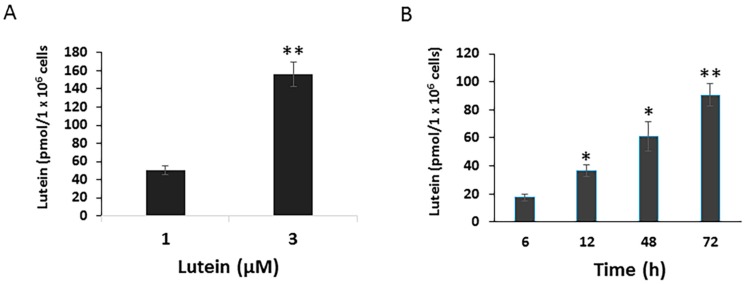
Dose and time-dependent cellular uptake of lutein in ARPE-19 cells. Cells were plated on six-well plates to reach confluence and then incubated with lutein at 1 or 3 μM for 24 h. After incubation, cells were analyzed for their carotenoid content by HPLC analysis. Values are expressed as picomoles of carotenoid per million of cells (**A**) Data are shown as means ± SD of three independent experiments. **: *p* < 0.001 compared with lutein at a given concentration. (**B**) Time course of lutein uptake in ARPE-19 cells. Cells were incubated with lutein at 1 μM for varying times (6 h up to 72 h). After incubation, cells were analyzed for their lutein content by HPLC analysis. Data are shown as means ± SD of two independent experiments, *: *p* < 0.05, **: *p* < 0.001.

To determine potential mechanisms by which lutein protects the RPE from environmental damage, we first investigated the uptake of lutein by cultured RPE cells. ARPE-19 cells, cultured with normal DMEM/F12, contained no detectable lutein or zeaxanthin (data not shown). When cells were incubated with 1 μM lutein for 24 h, the concentration of lutein in the cells rose to 50.6 pmol/1 × 10^6^ cells ± 4.87 pmol/1 × 10^6^ cells. Cellular lutein uptake increased in parallel to the increased lutein concentration. After 24 h incubation with 3 μM lutein, the cellular lutein levels reached to 156.3 pmol/1 × 10^6^ cells ± 13.56 pmol/1 × 10^6^ cells (Figure 1A). Moreover, RPE lutein uptake was a time-dependent process. As shown in Figure 1B, the uptake of lutein by ARPE-19 significantly increased in a time-dependent manner.

### 3.2. Determination of the Expression of Genes Involved in Xanthophyll Uptake, Metabolism and Transport in ARPE-19 Cells

**Figure 2:**
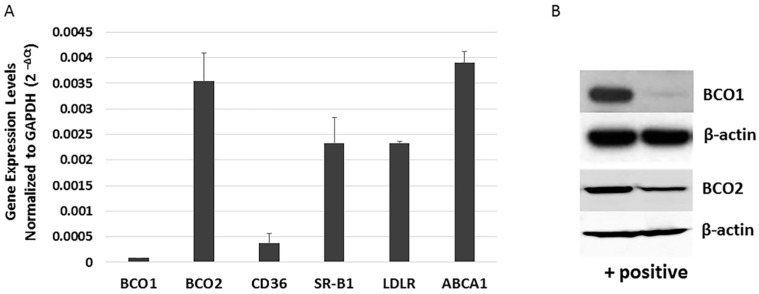
Expression of xanthophyll uptake-, metabolism- and transport-related genes in ARPE-19 cells. (**A**) mRNA levels of selected genes related to xanthophyll uptake (SR-BI, LDLR and CD36), metabolism (BCO1 and BCO2) and transport (ABCA1) in undifferentiated ARPE-19 cells were determined by qRT-PCR. (**B**) Western blot analysis verifying the difference in BCO1 and BCO2 expression. Left lane indicates (+) control cells (transfected HERK293); right lane shows ARPE-19 cells.

The human retina and RPE express varying amounts of carotenoid cleavage enzymes (BCO1 and BCO2), transport related protein (ABCA1), and scavenger receptors (SR-B1, CD36, and LDLR) [20]. BCO1 mRNA and protein has been detected in the human RPE cell line D407 [21], but no study has investigated BCO2 expression in more commonly utilized human RPE cell lines. To better understand the role of BCO1, BCO2, and xanthophyll uptake- and transport-related genes in the RPE, we re-evaluated the expression of BCO1, BCO2, and xanthophyll metabolism-related transcripts in ARPE-19 cells using quantitative PCR (qRT-PCR). As shown in Figure 2A, ARPE-19 cells robustly expressed BCO2, SR-B1, and LDLR, and lower levels of BCO1 and CD36. In a similar fashion, BCO2 protein was more readily detectable in ARPE-19 cells than is BCO1 (Figure 2B).

### 3.3. Effects of Selected Carotenoids on the Expression of BCO1, BCO2, and Scavenger Receptors in ARPE-19 Cells

**Figure 3:**
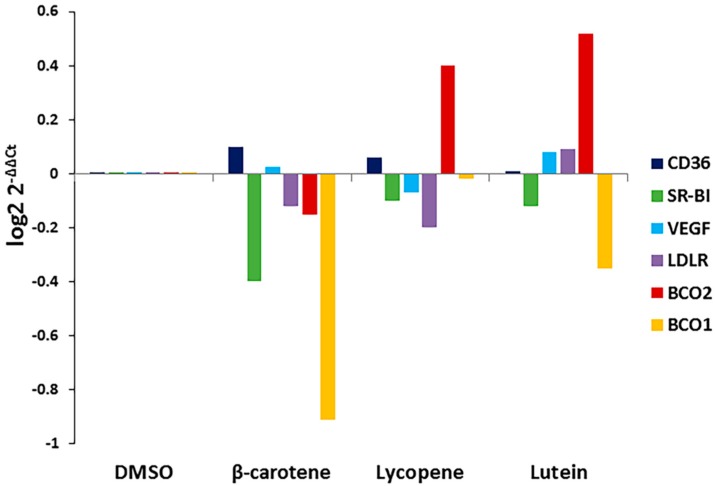
Effects of specific carotenoids on the expression of selected xanthophyll metabolism-related genes in ARPE-19 cells. ARPE-19 cells were treated with 1 μM concentration of indicated carotenoids for 24 h. Total RNA was isolated and qRT-PCR was performed for indicated genes. Data are shown as expression of fold changes (log_2_ 2^−ΔΔCt^).

Carotenoid substrate availability regulates the expression of BCO1 and BCO2 in other tissues. To determine whether the addition of carotenoids affects the expression of BCO1, BCO2, or xanthophyll uptake-related genes in the RPE, we treated cells with the three carotenoids and determined effects on BCO1, BCO2, VEGF, and scavenger receptor (SR-B1, CD36, LDLR) gene expression. As shown in Figure 3, exposure to specific carotenoids (β-carotene, lycopene, and lutein) altered ARPE-19 gene expression patterns. The expression of BCO1 and SR-B1 was significantly decreased in cells treated with lutein and β-carotene when compared to untreated control; lycopene and lutein dramatically induced the expression of BCO2.

### 3.4. Effects of Carotenoids on RPE Cell Growth

**Figure 4:**
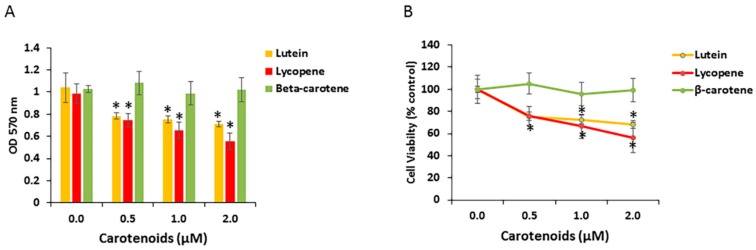
Effects of carotenoids on cell growth in ARPE-19. (**A**) Lutein and lycopene, but not beta-carotene, inhibited cell growth in undifferentiated APRE-19 cells. ARPE-19 cells were treated with indicated concentrations of carotenoids (0.5–2 μM) for 48 h. Cell viability was measured by MTT assay. Data are shown as means ± SD of three different experiments (*n* = 3). *, *p* < 0.05. (**B**) Inhibition of ARPE-19 cell growth in the presence of indicated concentration of carotenoids. Cell viability was expressed as percentage of control. Data are shown as means ± SD of three different experiments (*n* = 3). *, *p* < 0.05.

Certain carotenoids have anti-proliferative activity in a variety of cell types. To examine RPE cell growth response to specific carotenoids, we investigated the effect of the three carotenoids (lutein, β-carotene, lycopene) on the proliferation of human ARPE-19 cells. Cells were treated with a physiological concentration of carotenoids (0.5 to 2 μM) for 48 h. Cell viability/growth was monitored with the MTT assay and normalized to untreated cells. As shown in Figure 4A, when compared with vehicle control, ARPE-19 cell growth was significantly decreased after 48 h exposure to lutein or lycopene, but to not β-carotene. However, the reduction in cell numbers was not clearly dose-dependent in this concentration range (Figure 4B). In several experiments, we also examined effects of the lutein isomer zeaxanthin on RPE cell numbers. Zeaxanthin exposure induced similar effects to lutein on an equimolar basis (data not shown). We did not study meso-zeaxanthin, a retinal metabolite of lutein.

### 3.5. Effects of Carotenoids on RPE Cell Growth under Hypoxic Conditions

**Figure 5:**
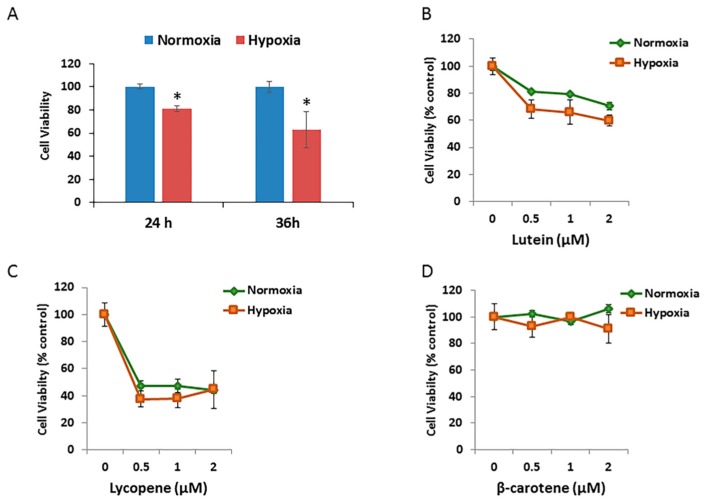
Effects of hypoxia and carotenoids on Retinal pigment epithelial (RPE) cell growth. (**A**) Comparison of ARPE-19 cell survival exposed to normoxic and hypoxic conditions for 24 h and 36 h. Cell viability was measured by MTT assay and expressed as percentage of normoxic condition. Data are shown as means ± SD of three different experiments (*n* = 3). *, *p* < 0.05. (**B**) ARPE-19 cells were pretreated with the indicated concentration of lutein for 12 h, followed by exposure to either normoxic or hypoxic conditions for 36 h. Data are shown as means ± SD of three different experiments (*n* = 3). (**C**) Pretreatment of lycopene ± hypoxia. (**D**) Pretreatment of β-carotene ± hypoxia.

To test effects of different oxidative stressors on the RPE cell growth, ARPE-19 cells exposed to hypoxic condition for different durations were assessed using MTT assays. As shown in Figure 5A, the mean relative cell viability decreased to 81.0% after 24 h of incubation and to 63.1% after 36 h of incubation under 1% O_2_ (hypoxia) compared with that under 21% O_2_ (normoxic) atmosphere.

The effects of different concentrations of three carotenoids on the cell growth/viability of hypoxic RPE cells were then investigated. The relative cell viability/growth significantly decreased by 15% after 24 h of incubation in hypoxic cells treated with 0.5 μM to 2.0 μM of lutein, compared with that in the normoxic group (Figure 5B). There were no significant changes in cell viability after 24 h of incubation in either the normoxic or hypoxic groups treated with lycopene (Figure 5C) or β-carotene (Figure 5D).

### 3.6. Effects of Lutein and Lycopene on Oxidative Stress-Induced RPE Cell Death

**Figure 6:**
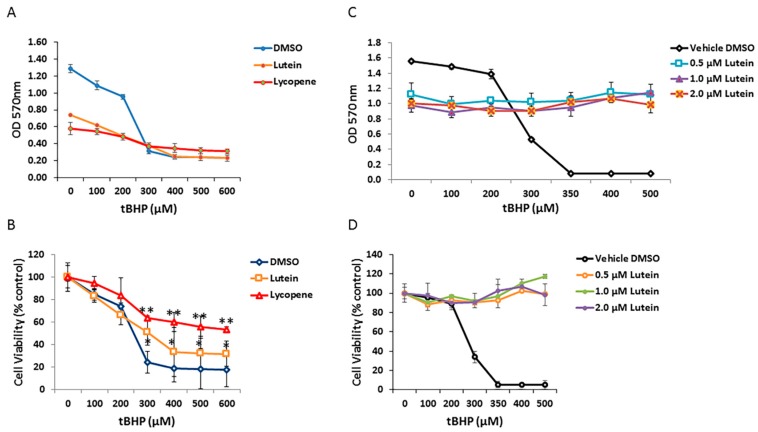
Protective effects of lutein and lycopene against oxidative stress-induced cell death. (**A**) ARPE-19 cells pretreated with vehicle control and 1 μM concentration of each carotenoids were exposed to different concentrations of *tert*-butyl hydroperoxide (tBHP). Cell survival was evaluated by MTT assay. (**B**) Analysis of lutein and lycopene protective effect on RPE cells under different tBHP concentrations. Cell viability was normalized to non-treated controls and was expressed as percentage of control. Data are shown as means ± SD of three different experiments (*n* = 3). *, *p* < 0.05; **, *p* < 0.001. (**C**) Comparison of ARPE-19 cell survival upon co-treatment of lutein and tBHP. (**D**) Analysis of protective effect of co-exposure of lutein and tBHP on RPE cells.

To determine if carotenoids can protect human RPE cells from oxidative stress induced by tBHP, ARPE-19 cells were pretreated with 1 µM of selected carotenoids (lycopene and lutein) for 12 h and then exposed to graded concentrations of tBHP for 24 h. As shown in Figure 6A, cell viability of ARPE-19 cells was dramatically decreased in the no-carotenoid-added control, in the presence of 300 µM tBHP. In contrast, lutein and lycopene pretreatment appeared to rescue RPE cells from tBHP-induced oxidative stress and cell death; lutein and lycopene pretreated cells showed increased survival compared with cells exposed to tBHP without lutein or lycopene pre-incubation (Figure 6B). To test whether lutein can directly neutralize oxidants by intracellular redox reactions, we added 0, 0.5, 1.0, and 2.0 µM lutein to RPE cells at the time of oxidative stress. Under these conditions, lutein completely protected RPE cells from tBHP-induced cell death (Figure 6C,D), suggesting that it has a direct antioxidant effect.

## 4. Discussion

The RPE cellular monolayer, interposed between the retinal microcirculation and neuroretinal/photoreceptor layers, is essential for maintaining retinal integrity and photoreceptor survival. The RPE is exposed to a host of physiological stressors, including light, hyperoxia, reactive oxygen species (ROS) generation, changes in blood glucose, and, in disease, ischemia and hypoxemia. In humans, the RPE contains the molecular pathways for the visual (retinoid) cycle and uptake, metabolism, and transfer of dietary lutein and its isomer zeaxanthin. Low or decreased retinal lutein/zeaxanthin levels, comparted to the normal eye, are found in both ischemic retinopathies and AMDs [5]. In vivo, the RPE maintains continuous contact with the photoreceptor layer and transports visual cycle retinoids, xanthophylls, nutrients, and photoreceptor debris. Several studies indicate that the scavenger receptor SR-B1 is involved in lutein and zeaxanthin uptake [20,22,23], suggesting a facilitated process. Circulating xanthophylls are associated principally with plasma high density lipoprotein (HDL) and are presented to the RPE membrane presumably, at least in part, by SR-B1 and CD36. In the present study, using relatively undifferentiated ARPE-19 cells, we provide evidence that in cell culture, lutein is taken up in a time- and concentration-dependent manner. In cell culture systems, a passive diffusion mechanism or a facilitated process could be involved in the cellular uptake of lutein. Recent studies of lutein transport have revealed that scavenger receptors (SR-B1, SR-B2, and CD36) variously contribute to lutein uptake [24]. Our current study demonstrates that ARPE-19 cells express relatively high levels of SR-B1 and LDLR, suggesting that cellular uptake of lutein in ARPE-19 cells may be a facilitated process. The ARPE-19 cell line has been used as a frequent in vitro model for the investigation of cellular functions of human RPE [25]. Since RPE cells are responsible for clearance of oxidants derived from photoreceptor turnover, ARPE-19 cells have been used to explore oxidant-mediated insults and related protective factors in various studies [26,27,28,29,30,31,32]. However, the ARPE-19 cell line does not exactly mimic RPE in vivo. Moreover, whether these cells are maintained in relatively undifferentiated or differentiated, polarized conditions, alters responses to carotenoid treatment and oxidative stress insults [33]. We show that undifferentiated ARPE-19 cells are more sensitive to oxidative stress than differentiated cells.

In the present study, we compared the effects of three carotenoids on changes in ARPE-19 cell viability/growth under conditions of normoxia, hypoxia, and oxidative stress. The novel finding is that lutein and lycopene, but not beta-carotene, inhibit cell growth. Lutein and lycopene have previously been described as antiproliferative agents in various types of cancer cells [34,35]. The present study extends these findings, showing that lutein and lycopene inhibit proliferation of undifferentiated RPE cells. We found no evidence that beta-carotene, in this “physiological” concentration range, inhibited RPE cell proliferation, in contrast to previous findings showing such inhibition in RPE cells [36]. Both lutein and lycopene have been found to inhibit RPE cell migration, which occurs in proliferative retinal diseases, such as proliferative vitreoretinopathy (PVR) and proliferative diabetic retinopathy (PDR) [37,38]. These inhibitory effects of lutein and lycopene may mitigate diseases resulting from RPE cell proliferation, including PVR, PDR, and AMD. Interestingly, lutein and lycopene are substrates for the enzyme BCO2. We show that BCO2 is expressed in these RPE cells and its expression is increased by exposure of cells to either carotenoid substrate, but not to β-carotene (Figure 3). This finding raises the possibility that at least some lutein- and lycopene-induced cellular effects may be due to carotenoid metabolism in the RPE.

The pathological feature of retinal ischemia is shared by many retinal diseases, including diabetic retinopathy (DR), ischemic retinal-vein occlusion, and retinopathy of prematurity (ROP). At the cellular level, ischemic injury includes oxidative damage caused by increased energy failure and hypoxia [39]. Tissue hypoxia is a consequence of many diseases in the retina, such as AMD, DR, and ROP. As such, strategies aimed at mitigating hypoxia-induced retinal cell damage may be important for restoring a more normal retinal environment and preventing retinal injury. In our study, we were not able to detect significant differences in the cellular responses to the carotenoids (lutein, lycopene, and beta-carotene) tested during hypoxia-induced injury in ARPE-19 cells. 

Numerous studies have shown that lutein/zeaxanthin is an ROS scavenger in extracellular environments [40] and, in several cell types, it has a protective effect against oxidative stress-induced cell damage [16,32]. In the present study, we demonstrate that lutein and lycopene reduce tBHP-induced cell death in ARPE-19 cells. These data extend the knowledge that lutein (and lycopene) may have protective roles in prevention of retinal diseases, in which oxidative damage plays an important pathogenetic role. Due to the unique location of the RPE, this cell layer is particularly subject to accumulation of ROS, which, in turn, leads to mitochondrial dysfunction and cell death [41,42]. Regarding the possible mechanism by which lutein (and lycopene) decrease tBHP-induced RPE cell death, our study indicates that these carotenoids may have direct antioxidative effects by scavenging intracellular ROS. Of note, oxidative stress may be a key contributor to RPE dysfunction in several retinal diseases. We selected tBHP as a pro-oxidant stressor because tBHP has been reported to induce lipid peroxidation, a self-propagating form of oxidative injury that damages cell membranes and presents a particular risk to the RPE and lipid-rich photoreceptor cells. In our experiments, tBHP treated cells showed decreased cell viability (Figure 6.). Lutein or lycopene pretreatment significantly increased cell survival after tBHP incubation, consistent with the observation that lutein and lycopene lower intracellular ROS production in tBHP-treated ARPE-19 cells [43,44]. The antioxidant and protective effects of lutein and lycopene have been well-documented by in vitro and in vivo studies [7,16,45,46,47,48,49]. Interestingly, and consistent with our findings, lutein and lycopene prevent ARPE-19 cell death caused by oxidative stress by reducing both intracellular and extracellular ROS production and, thus, confer a protective effect against oxidative stress-induced cell damage.

## 5. Conclusions

In conclusion, this study provides evidence that lutein and lycopene protect ARPE-19 cells from tBHP-induced oxidative damage and this protective effect may be associated, at least partly, with direct antioxidant scavenging of ROS. We demonstrate, for the first time, antiproliferative effects of lutein and lycopene in ARPE-19 cells, suggesting that lutein (and lycopene, if presented in sufficient concentrations) may contribute to the prevention or mitigation of RPE proliferative diseases, such as PVR, ROP, and AMD. Our results demonstrate that lutein and lycopene have robust protective effects against oxidative damage to RPE cells.
